# 

**Published:** 1893-09-30

**Authors:** 


					September 3Ct
WITH EXTRA NURSING SUPPLEMEN
Per Annum, 8s. 6d., post free.
I Monthly Parts, 6d.; post free, 8d.
Ditto per Annum, 8s., post free.
No. 366^ Vol. XIY. Saturday,
September 30th, 1893.
[Twopence.

				

